# A Lightweight Localization Strategy for LiDAR-Guided Autonomous Robots with Artificial Landmarks

**DOI:** 10.3390/s21134479

**Published:** 2021-06-30

**Authors:** Sen Wang, Xiaohe Chen, Guanyu Ding, Yongyao Li, Wenchang Xu, Qinglei Zhao, Yan Gong, Qi Song

**Affiliations:** 1 School of Electronic and Information Engineering, Changchun University of Science and Technology, Changchun 130022, China; 2019100549@mails.cust.edu.cn (S.W.); liyongyao@mails.cust.edu.cn (Y.L.); 2 Suzhou Institute of Biomedical Engineering and Technology, Chinese Academy of Sciences, Suzhou 215163, China; chenxh@sibet.ac.cn (X.C.); xuwc@sibet.ac.cn (W.X.); gongy@sibet.ac.cn (Y.G.); 3 Pilot AI Company, Hangzhou 310000, China; dingguanyu@gmail.com; 4 Changchun Institute of Optics, Fine Mechanics and Physics, Chinese Academy of Sciences, Changchun 130033, China; coldsun@sina.com

**Keywords:** LiDAR navigation, reflector localization, motion compensation, reflector matching, high-speed movement

## Abstract

This paper proposes and implements a lightweight, “real-time” localization system (SORLA) with artificial landmarks (reflectors), which only uses LiDAR data for the laser odometer compensation in the case of high-speed or sharp-turning. Theoretically, due to the feature-matching mechanism of the LiDAR, locations of multiple reflectors and the reflector layout are not limited by geometrical relation. A series of algorithms is implemented to find and track the features of the environment, such as the reflector localization method, the motion compensation technique, and the reflector matching optimization algorithm. The reflector extraction algorithm is used to identify the reflector candidates and estimates the precise center locations of the reflectors from 2D LiDAR data. The motion compensation algorithm predicts the potential velocity, location, and angle of the robot without odometer errors. Finally, the matching optimization algorithm searches the reflector combinations for the best matching score, which ensures that the correct reflector combination could be found during the high-speed movement and fast turning. All those mechanisms guarantee the algorithm’s precision and robustness in the high speed and noisy background. Our experimental results show that the SORLA algorithm has an average localization error of 6.45 mm at a speed of 0.4 m/s, and 9.87 mm at 4.2 m/s, and still works well with the angular velocity of 1.4 rad/s at a sharp turn. The recovery mechanism in the algorithm could handle the failure cases of reflector occlusion, and the long-term stability test of 72 h firmly proves the algorithm’s robustness. This work shows that the strategy used in the SORLA algorithm is feasible for industry-level navigation with high precision and a promising alternative solution for SLAM.

## 1. Introduction

Autonomous mobile robots (AMRs) can significantly release manpower from heavy fetching tasks and boost efficiency and avoid human error from repeatable operations [1]. In particular, with the development of LiDAR-based navigation techniques, mobile robots could be located in “real time” in complex environments, and accurate localization is highly desired to ensure the performance and safety of autonomous mobile robots [2,3]. Navigation technology is one of the fundamentals in the field of automation and robotics. Lots of research activities and industry applications are conducted with Laser SLAM, inertial navigation, magnetic tapes, and Visual-SLAM techniques. Not like the typical SLAM approach, the laser-based reflector localization technique is the classic solution and has the advantages of high-precision localization, high-speed processing, and decent robustness with no accumulated errors. For example, automated guided vehicles (AGVs) and industrial forklift robots in the warehouse are the cases with the highest demand for the automation. The stringent requirements of localization make LiDAR-based reflector localization the best candidate so far.

Localization of mobile robots refers to the process in which mobile robots estimate their positions and pose angles through sensors’ perception techniques [4,5]. Recent progress in autonomous vehicles and LiDAR devices has dramatically reduced the cost of LiDAR hardware and pushed the LiDAR-based SLAM to become the most promising approach for self-driving vehicles and robots with great flexibility [6,7,8,9]. Laser-based SLAM can provide centimeter-level precision and does not need any extra modification of the environment [10]. However, the ability of mapping is still limited to the low-speed applications in a small and “feature-rich” environment. For unmanned vehicles or robots, the warehouse environment lacks the so-called “features”, and most scenes are quite similar from robot’s perception, which is one of the main obstacles causing navigation failure [11,12]. Many studies have been conducted about the sensor fusion technique, and Visual-SLAM is an alternative approach for 3D SLAM with low-cost camera [13], but it is gated by the computing capability. Another approach is the LiDAR-based localization technique assisted with artificial landmarks, which refers to the cylindrical reflectors in this study. Different from laser SLAM, which can realize localization and mapping simultaneously, the LiDAR-based reflector localization algorithm (RLA) requires the pre-installation of reflectors to provide enough “features” to help the localization process. Since RLA uses the static map as the localization reference, it has the characteristics of high localization accuracy, high stability, and high resistance to noise. Because the information of artificial landmarks can be clearly identified and processed referring to the existing map [14], there will be no cumulative error in the algorithm, which is a large advantage for long-term operation and highly desired for the industry applications.

Even artificial landmarks have been used in laser navigation for decades. There is still much research work ongoing about investigating novel functions and features for efficient matching and remarkably reducing the computation workload. The localization technique based on the trilateration method utilizes the adaptive unscented Kalman filter method to improve the accuracy of localization [15]. The tracking and localization algorithm are proposed to improve positional accuracy with the optimal triangular positioning method [16]. Farouk Ghallabi presented a localization algorithm by matching road perceptions from a 3D LIDAR sensor with HD map elements. This method estimates the position of the vehicle by matching the observed HRL with the HD map attributes [17]. Davide Ronzoni provided the algorithm for AGV self-localization based on landmark identification to solve the global localization problem for an industrial AGV moving in a known environment [18].

In this paper, we propose a “light-weight” reflector localization algorithm (SORLA) using 2D LiDAR data and the reflective cylinders as landmarks with precision of up to 6.45 mm. To the best of our knowledge, this is one of the most precise results in the field of industry-level autonomous robots. LiDAR-based localization with artificial landmarks is a relatively mature methodology for the industrial robotic applications [19,20]. It utilizes a “well-designed” 2D feature map to help improve localization precision and algorithm robustness. The feature map is composed with artificial landmarks, which are the highly reflective geometrical objects in 2D. The approach proposed in this work uses LiDAR-based data only to locate the robot without IMU’s association [21,22,23,24], which also isolates the impact of sensor noise [25]. With the help of the reflector matching mechanism, the estimated odometer information is used to track and predict the location under high speed [26]. The details will be discussed in detail in Section 2.4.

## 2. Materials and Methods

### 2.1. System Description

#### 2.1.1. The Building of the Coordinate System

**Figure 1:**
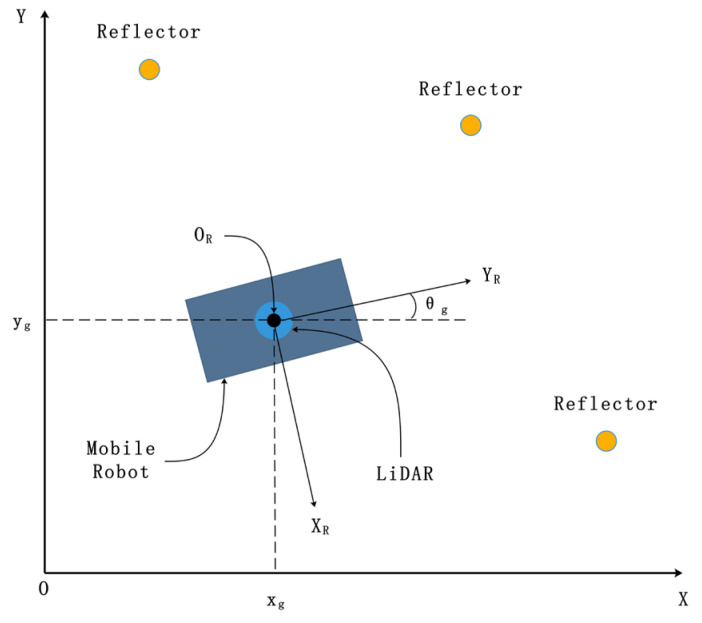
The coordinate system description.

For the generality of setup, LiDAR is installed at the center of the robot, which means that the position of LiDAR can be used to represent the position of the robot in a two-dimensional configuration. In this research, two coordinate systems are established respectively: the global coordinate system OXY and the robot coordinate system $O_{R}X_{R}Y_{R}$ centered on the mobile robot, where the center of the robot coordinate system is $O_{R}$ (Figure 1).

The LiDAR device will obtain a full frame of scanning data set from each rotation as $\left\{(d_{j},\theta_{j}),\sigma_{j}|j=1,2,\ldots ,n\right\}$, where $d_{j}$ is the linear distance between the *j*-th data point from the LiDAR center, $\theta_{j}$ is the azimuth angle of the *j*-th data point in the polar coordinate system centered on LiDAR, and $\sigma_{j}$ is the reflection intensity value of the *j*-th data point. The x and y coordinate of the *j*-th data point in the robot coordinate system is:

(1) $$\left\{ \begin{array}{l} Rx_{j}=d_{j}\cos(\theta_{j})\\ Ry_{j}=d_{j}\sin(\theta_{j}) \end{array}\right.$$

Suppose that the robot’s pose in the global coordinate system is $\left(x_{g},y_{g},\theta_{g}\right)$, where $x_{g}$ and $y_{g}$ is the position of the robot coordinate system with center $O_{R}$ in the global coordinate system, and $\theta_{g}$ is the rotation angle of the robot coordinate system in the global coordinate system.

#### 2.1.2. LiDAR-Based Localization System

**Figure 2:**
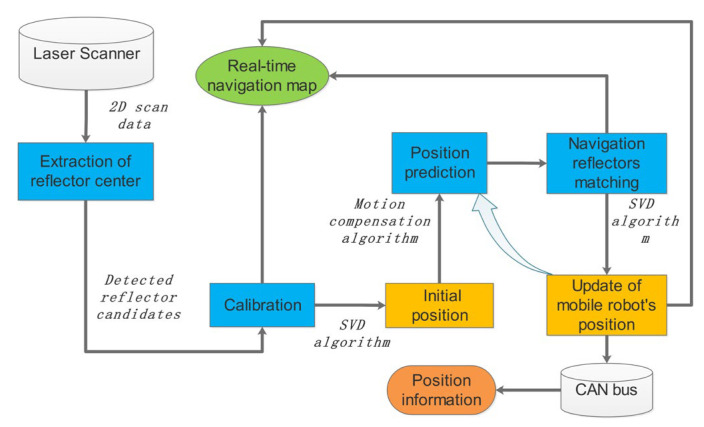
“SORLA” program architecture diagram.

A series of cylindrical reflectors with high reflectivity are installed in the actual warehouse environment. LiDAR is installed on the top of the mobile robot to collect the scanning data of the surrounding environment, avoiding any occlusion. The navigation system consists of the “SORLA” program (Figure 2), a Pepper-Fuchs R2000 Laser Scanner, and a CAN bus device. The CAN bus device is used to receive the control messages and send out localization data messages in the specific format.

When the robot is moving at a high speed, the SLAM algorithm is prone to odometry errors. One solution is to correct the errors by using extra sensors such as IMU, However, this will require extra hardware with the calibration process and will increase integration cost. The advantage of the navigation system in this study is that, due to the use of motion compensation algorithms and reflector sequencing optimization, the system only needs LiDAR data to suppress the odometry error caused by the acceleration or deceleration.

Here, we define two modes: initialization mode and navigation mode. The location of the reflectors used in the initialization mode and the navigation mode are stored in the “initialization reflector map” and the “navigation reflector map”, respectively. The advantage is that the reflector layout of the navigation mode does not rely on the geometric relationship to determine the location, as the initialization mode does. The current pose can be estimated from the “last-step” localization result and the motion compensation algorithm. Firstly, the initial position and orientation can be acquired in the initialization mode, then navigation mode can be performed subsequently to track the location changes in real-time; finally, the position of the mobile robot in the global coordinate system is calculated by the SVD algorithm [27,28].

**Figure 3:**
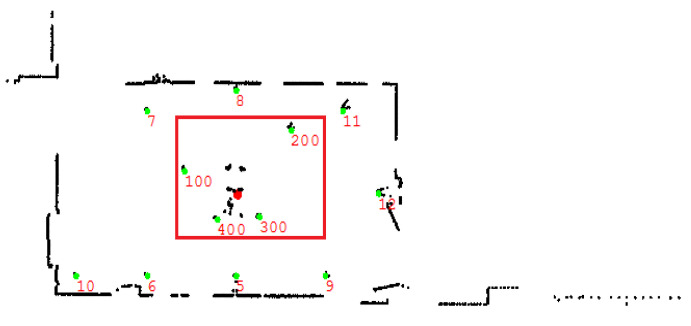
Real-time navigation map.

The navigation system establishes a 2D real-time navigation map (Figure 3) composed of the reflectors by processing the scan data of the laser scanner on the surrounding environment. In order to obtain the location and orientation information independently, the geometrical relation of the reflectors in the initialization mode has to be easily distinguishable. The area in the red rectangle is the initialization area. The red dot represents the “real-time” position of the mobile robot, each green dot represents the “ideal position” of the reflectors from the reflector map, and the black dots around them are the real time data from the laser scanner. We call the reflector used for initialization mode the “initialization reflector” and the reflector used for navigation mode the “navigation reflector”.

### 2.2. Extraction Algorithm of the Reflector Center Position

The extraction of the center point of the reflector is an important prerequisite of the reflector matching. Our analysis indicates that the accuracy of the reflector center point has a significant impact on the final localization accuracy of the system. The extraction algorithm is composed of three steps: (i) the extraction of the raw reflector data, (ii) the clustering of the reflector data, and (iii) the estimation of the reflector center. For the convenience of the following description, we call the reflector data in the initialization reflector map and navigation reflector map the “referential reflector data” and the reflector data in the actual site extracted by the reflector center extraction algorithm the “detected reflector data”.

#### 2.2.1. The Extraction of the Raw Reflector Data

Because of the high reflectivity of the reflectors, the returned light intensity from the reflectors is much stronger than other objects and the background. Therefore, the raw reflector data can be simply picked up from the laser scanning data by selecting a proper light intensity threshold. Suppose the LiDAR frame contains n data points: $\left\{M_{j}=(d_{j},\theta_{j}),\sigma_{j}|j=1,2,3,\ldots ,n\right\}$. In order to quickly and accurately distinguish the reflector data from the background, only the data of which the distance is greater than or equal to $d_{\min}$ are extracted; by filtering the reflection intensity, m raw reflector data points are selected $\left\{M_{k}=(d_{k},\theta_{k}),\sigma_{k}|k=1,2,3,\ldots ,m|1\leq k\leq m\right\}$. When the data points in $M_{j}$ meet the following conditions, they are considered the raw reflector data points:

(2) $$\left\{ \begin{array}{l} d_{k}\geq d_{\min}\\ \sigma_{k}\geq \sigma_{\min}\\ \sigma_{k}=\max\left\{\sigma_{p-1},\sigma_{p-1},\sigma_{p-1}|p=2,3,4,\ldots n-1\right\} \end{array}\right.$$

Here, $\sigma_{\min}$ is the threshold of the targeted reflection intensity, and $d_{\min}$ is the minimum distance to distinguish a reflector.

#### 2.2.2. Clustering of the Reflector Data

**Figure 4:**
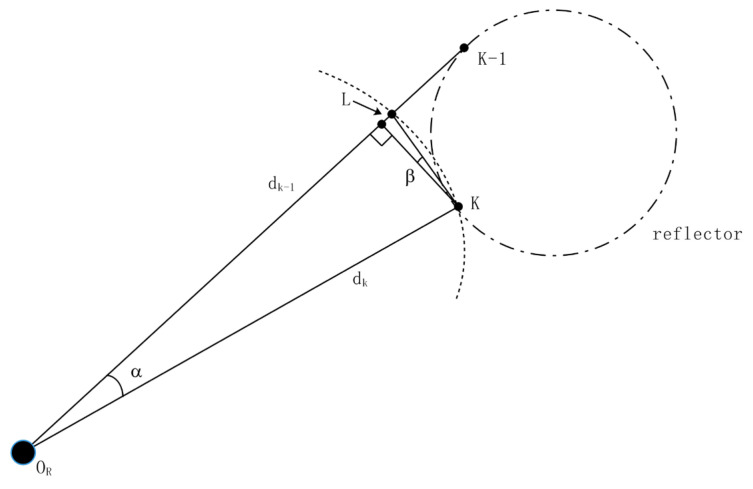
Calculation method of distance threshold D_k_ between the adjacent data points.

Practically, there are always some noise signals in the background of the data filtered by the reflection intensity. Therefore, it is very necessary to group the raw reflector data into the reflector clusters, and each cluster represents all data points from one single reflector. For example, if the distance and angle differences of two points are less than the threshold values, the two points can be from the identical reflector. In this step, the distance threshold *D_k_* and the angle threshold *φ_k_* of adjacent data points are set, and the diameter of each reflector is *R_0_*. Assuming that the raw reflector data are divided into q reflector data sets by the clustering method, the algorithm passes through the following constraints to traverse and compare the distance and angle of the adjacent raw reflector data points to obtain the data set belonging to the same reflector:

(3) $$\left\{ \begin{array}{l} \left|d_{k}-d_{k-1}\right|\leq D_{k}\\ \left|\theta_{k}-\theta_{k-1}\right|\leq \varphi_{k} \end{array}\right.,$$

where $\varphi_{k}=\left\{\frac{180^{0}R_{0}}{\pi d_{k}}|k=2,3,4\ldots m\right\}$,$d_{k}<d_{k-1}$, the angle between the adjacent points and the center line of the LiDAR is *φ*. A vertical point is set on the longer distance $d_{k-1}$ to introduce L (Figure 4). It is not hard to work out from the simple geometric relation. β=α/2, $L=\tan\beta \cdot d_{k}\cdot \sin\alpha$. Therefore, it can be concluded that:

(4) $$D_{k}=L=\tan(\alpha /2)\cdot d_{k}\cdot \sin\alpha .$$

#### 2.2.3. The Estimation of the Reflector Center

**Figure 5:**
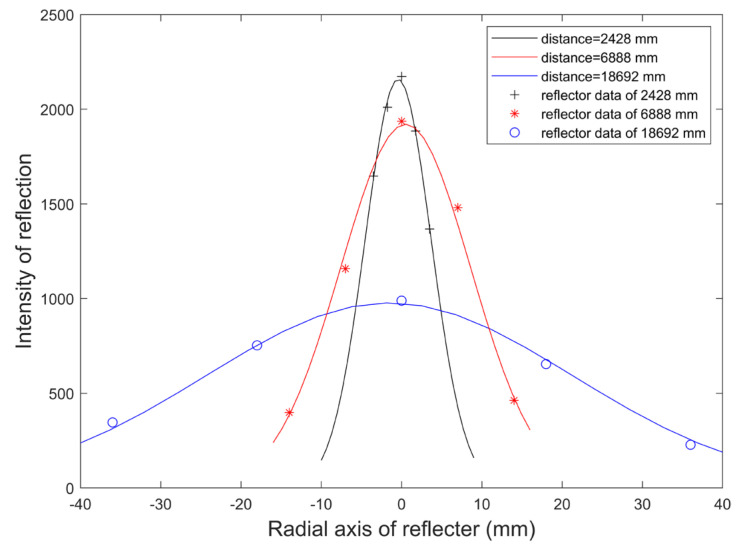
Fitting results of Gaussian function on the reflection intensity of the reflector data at different distances.

Since the reflector intensity profile from a single reflector cluster shows a nonlinear distribution, a Gauss fitting technique [29] is used to locate the center peak location, which also can be used to present the highest possible location of the reflectors. In this section, the center position of the reflector is estimated by fitting the peak value of the reflection intensity with the Gaussian function. In Figure 5, the reflected intensity profile at the closer location decreases more sharply from the center peak, while the reflection intensity profile at the larger distance shows a flattened trend. By applying adaptive parametric fitting, the Gaussian curve is able to appropriately present the center of the reflectors. Therefore, the reflection intensity $\sigma_{f}$ of the central point in the reflector data set $M_{r}$ can be obtained. Suppose there are s data points in the *r*-th reflector data set, forming a collection $S_{1}=\{0,1,2\ldots ,s\}$, $u\in S_{1}$. Then, the reflection intensity of the *r*-th reflector data set $\sigma_{r}$ can be obtained by polynomial fitting:

(5) $$\log(\sigma_{r})=p_{1}u^{2}+p_{2}u+p_{3}.$$

The coefficients of the Gaussian fitting model are further determined:

(6) $$a=e^{\left(p_{3}-\frac{p_{1}^{3}p_{2}^{2}}{4}\right)},b=-\frac{p_{1}p_{2}}{2},c=\sqrt{-\frac{1}{p_{1}}}.$$

Divide $S_{1}$ into 4s elements to get $S_{2}$,$w\in S_{2}$, then the fitting reflection intensity $\sigma_{g}$ corresponding to the element in $S_{2}$ obtained by Gaussian fitting model is as follows:

(7) $$\sigma_{g}=ae^{\left(-(\frac{{\left(w-b\right)}^{2}}{c})\right)}.$$

Thus, $\sigma_{f}=\max(\sigma_{g})$, and the index of the data corresponding to $\sigma_{f}$ is $I_{f}$. Let the first point in the *r*-th reflector data set be $\left(d_{1},\theta_{1},\sigma_{1}\right)$, the last point is $\left(d_{end},\theta_{end},\sigma_{end}\right)$, an angle threshold value $\theta_{\lambda }$ is set to determine the angle $\theta_{f}$ of the center point of the *r*-th reflector data point set, where $\theta_{\lambda }$ is:

(8) $$\theta_{\lambda }=\frac{(\theta_{end}-\theta_{1})I_{f}}{4s},$$

where $\theta_{f}=\theta_{1}+\theta_{\lambda }$. The point with the largest reflection intensity in the *r*-th reflector data set $\left(d_{m},\theta_{m},\sigma_{m}\right)$ is found, the index of the corresponding data point is Im, the interval threshold $\partial =\min((s-I_{m}),(I_{m}-1))$ is set, and $d_{f}$ is the mean value of the distance between the data points in the interval $\left[(I_{m}-\partial ),(I_{m}+\partial )\right]$ of the *r*-th reflector data set:

(9) $$d_{f}=\frac{d(I_{m}-\partial )+\ldots +d(I_{m}+\partial )}{2\partial }.$$

From above, the coordinates of the center point of the *r*-th reflector data point collection in the robot coordinate system are calculated as $\left(x_{r},y_{r}\right)$, and this coordinate is used to represent the actual position of the *r*-th reflector extracted from the raw data of LiDAR scanning,

(10) $$\left\{ \begin{array}{l} x_{r}=d_{f}\cos(\theta_{j})\\ y_{r}=d_{f}\sin(\theta_{j}) \end{array}\right..$$

### 2.3. Reflector Matching Algorithm in Initialization Mode

Before navigation starts, it needs to obtain the initial location and angle of the robot by running the initialization mode, and the number of initialization reflectors is at least 3 to guarantee the initial localization calculation. The initialization reflector map is a set of reflector position coordinates used to obtain the initial position calculation. The design principle of the initialization reflector should be as follows. (1) The distance difference between any two reflectors should be greater than 300 mm, and (2) the angle difference between every two reflectors should be greater than $6^{\circ }$. In this step, the navigation system first finds three or more detected reflectors nearest to the mobile robot to form the candidate pool for the initial location calculation. The initial location of the mobile robot is obtained by matching the distance and angle value of the candidate pool with the referential reflector pool picked up from initialization reflector map by the simple searching mechanism.

It is assumed that there are N(N≥3) detected reflectors in the optimal matching area; the coordinate of the *r*-th detected reflector is $N_{r}=\left\{(d_{r},\theta_{r}),|r=1,2,\ldots ,N-1\right\}$, and M(M≥3) referential reflectors in the initialization reflector map; the coordinates of the *i*-th referential reflector are $M_{i}=\left\{(x_{i},y_{i})|i=1,2,\ldots ,M-1\right\}$. Then, the distance vector between each two detected reflectors and each two referential reflectors can be obtained, respectively:

(11) $$N_{dis\tan ce}=[d_{1,2},d_{1,3},\ldots d_{1,N},d_{2,3},d_{2,4},\ldots ,d_{2,N},\ldots d_{N-1,N}].$$

(12) $$M_{dis\tan ce}=[d_{1,2},d_{1,3},\ldots d_{1,M},d_{2,3},d_{2,4},\ldots ,d_{2,M},\ldots d_{M-1,M}].$$

According to the geometric principle, N(N−1)(N−2)/6 angles will be formed between any three of the N detected reflectors. Suppose that the linear distance between the *r*-th detected reflector and the (*r*-1)-th detected reflector is a, the one between the *r*-th detected reflector and the (*r* + 1)-th detected reflector is b, and the one between the (*r*-1)-th detected reflector and the (*r* + 1)-th detected reflector is c. The matching angle reference value $\theta_{r}$ between the three detected reflectors is represented by the angle value θ corresponding to the line c; we can obtain the following results:

(13) $$\theta_{r}=\theta =\arccos(\frac{a^{2}+b^{2}-c^{2}}{2ab}),$$

the angle vector between each three detected reflector and each three referential reflector can be acquired, respectively:

(14) $$N_{angle}=[\theta_{1},\theta_{2},\theta_{3},\ldots \theta_{\frac{N(N-1)(N-2)}{6}}].$$

(15) $$M_{angle}=[\theta_{1},\theta_{2},\theta_{3},\ldots \theta_{\frac{M(M-1)(M-2)}{6}}].$$

In order to make use of the above distance vector and angle vector to match the reflector quickly and accurately, we set the distance matching error threshold as $z_{f}$ and angle matching error threshold as $g_{f}$. The absolute value of the difference between each distance value of the distance vector $N_{dis\tan ce}$ between the detected reflectors and each distance value of the distance vector $M_{dis\tan ce}$ between the referential reflectors is calculated, and the distance difference matrix of reflector $Z_{dis\tan ce}$ is obtained:

(16) $$Z_{dis\tan ce}=\left[\begin{array}{ccccc} Z_{11} & Z_{12} & Z_{13} & \cdots & Z_{1N}\\ Z_{21} & Z_{22} & Z_{23} & \cdots & Z_{2N}\\ Z_{31} & Z_{32} & Z_{33} & \cdots & Z_{3N}\\ \vdots & \vdots & \vdots & \ddots & \vdots \\ Z_{M1} & Z_{M2} & Z_{M3} & \cdots & Z_{MN} \end{array}\right],$$

where $Z_{M,N}=\left|d_{M-1,M}-d_{N-1,N}\right|$.

Considering extreme small or large angles in the polygon are vulnerable to measurement error or noise, we only recognize the effective angle data between $\theta =5^{\circ }\sim 175^{\circ }$. Similarly, the absolute value of the difference between each angle value of the angle vector $N_{angle}$ between the detected reflectors and each angle value of the angle vector $M_{angle}$ between the referential reflectors are calculated, and the matrix of the angle difference of the reflector $G_{angle}$ is obtained:

(17) $$G_{angle}=\left[\begin{array}{ccccc} G_{11} & G_{12} & G_{13} & \cdots & G_{1N}\\ G_{21} & G_{22} & G_{23} & \cdots & G_{2N}\\ G_{31} & G_{32} & G_{33} & \cdots & G_{3N}\\ \vdots & \vdots & \vdots & \ddots & \vdots \\ G_{M1} & G_{M2} & G_{M3} & \cdots & G_{MN} \end{array}\right],$$

where $G_{MN}=\left|\theta_{\frac{M(M-1)(M-2)}{6}}-\theta_{\frac{N(N-1)(N-2)}{6}}\right|$.

Take the minimum value of each column in the distance difference matrix $Z_{dis\tan ce}$ and the angle difference matrix $G_{dis\tan ce}$, compared with $z_{f}$ and $g_{f}$ respectively. If $Z_{ij}$ in the distance difference matrix $Z_{dis\tan ce}$ is within $z_{f}$, and the distance between the *i*-th referential reflector and the *j*-th detected reflector is matched successfully, then $G_{ij}$ and $g_{f}$ are further compared. Therefore, the *i*-th referential reflector and the *j*-th detected reflector constitute a pair of matched reflector combinations.

### 2.4. Navigation Localization Algorithm

#### 2.4.1. Motion Compensation Algorithm

Unlike the initialization mode, the navigation mode uses a guess-and-matching strategy to make the guess on the “most-possible” reflector candidates and performs the matching calculation to obtain the location. When the robot moves with low speed, the travelling distance between two adjacent points is small, so the previous location can be directly used as initial guess location for the next round of calculation. However, with the high speed or fast turning angle speed, such an assumption will introduce a distance or angle error and cannot be ignored. This section proposes a motion compensation algorithm, which effectively eliminates the odometer error caused by high-speed movement.

The navigation system first uses a motion compensation algorithm to predict the real-time pose of the mobile robot during the movement to form a desired pose sequence. The estimated location is used to complete the navigation reflector matching in the navigation mode. Since the pose history of the robot from the previous moment is known, the pose and velocity estimates of the previous moment are used to predict the pose and speed of the current moment; each time the calculation is completed, the laser odometer will be recorded to form the robot’s trajectory and rotation trajectory histogram.

Because the scanning frequency of LiDAR is high, we assume that there is no significant change in the robot’s speed and angular velocity in a scanning period, and the scanning period of LiDAR is t. In order to obtain the key parameters of the robot motion model, suppose that the pose of the robot at the last moment is $\left(x_{p},y_{p},\theta_{p}\right)$, and the time period from time zero to the previous time is divided into 2N time periods according to the scanning period; correspondingly, the velocity trajectory and rotation trajectory of the robot from time zero to the previous time are also divided into 2N sub-trajectories according to the scanning period. Here, a digital averaging filter is used to smooth out the speed changes across adjacent N calculations. Then, the horizontal velocity $v_{x}$, vertical velocity $v_{y}$, and angular velocity $\theta_{v}$ at the current moment are:

(18) $$\left\{ \begin{array}{l} v_{x}=\frac{1}{Nt}\sum_{i=1}^{N}X_{1,i}\\ v_{y}=\frac{1}{Nt}\sum_{i=1}^{N}Y_{1,i}\\ \theta_{v}=\frac{1}{Nt}\sum_{i=1}^{N}\theta_{1,i} \end{array}\right.,$$

where $X_{1,i}$, $Y_{1,i}$ are the horizontal and vertical offsets corresponding to the *i*-th scan period (i≤N) in the (*N*~2*N*)-th sub-motion trajectory; $\theta_{1,i}$ is the angular offset corresponding to the i-th scanning period of the (*N*~2*N*)-th sub-rotation trajectory.

The x acceleration $a_{x}$, y acceleration $a_{y}$ and angular acceleration $a_{\theta }$ corresponding to the current moment are:

(19) $$\left\{ \begin{array}{l} a_{x}=\frac{1}{Nt}((\sum_{i=1}^{N}X_{1,i})-(\sum_{i=1}^{N}X_{2,i}))\\ a_{y}=\frac{1}{Nt}((\sum_{i=1}^{N}Y_{1,i})-(\sum_{i=1}^{N}Y_{2,i}))\\ a_{\theta }=\frac{1}{Nt}((\sum_{i=1}^{N}\theta_{1,i})-(\sum_{i=1}^{N}\theta_{2,i})) \end{array}\right..$$

where $X_{2,i}$, $Y_{2,i}$ are the *x* and *y* offsets corresponding to the *i*-th scan period (i≤N) in the (0~*N*)-th sub-motion trajectory; $\theta_{2,i}$ is the angular offset corresponding to the *i*-th scanning period of the (0~*N*)-th sub-rotation trajectory.

Then, the pose of the robot at the current moment estimated by the motion compensation algorithm is $\left(x_{e},y_{e},\theta_{e}\right)$:

(20) $$\left\{ \begin{array}{l} x_{e}=x_{p}+\frac{1}{N}\sum_{i=1}^{N}X_{1,i}\\ y_{e}=y_{p}+\frac{1}{N}\sum_{i=1}^{N}Y_{1,i}\\ \theta_{e}=\theta_{p}+\frac{1}{N}\sum_{i=1}^{N}\theta_{1,i} \end{array}\right..$$

Due to the fact that the reflector is prone to matching failure when the robot is accelerating or decelerating, the navigation system can perform compensation calculations on the position of the detected reflectors according to the motion compensation algorithm.

Suppose that the LiDAR scans *k* detected reflectors during movement, and these reflectors form a collection of detection reflectors $ref_{k}$. Among them, the coordinate of the *j*-th detected reflector in the robot coordinate system is $K_{j}=\left\{(x_{j},y_{j}),\sigma_{r}|j=1,2,3,\ldots ,k\right\}$; then, the position $\left(x_{jj},y_{jj}\right)$ after optimizing the position of the *j*-th detected reflector at the current moment by using the motion compensation algorithm is:

(21) $$\left\{ \begin{array}{l} x_{jj}=x_{j}-v_{x}\cdot t(k-j/k)\\ y_{jj}=y_{j}-v_{y}\cdot t(k-j/k) \end{array}\right..$$

#### 2.4.2. Reflector Matching Algorithm in Navigation Mode

Because the current pose can be estimated according to the motion compensation algorithm, the position of the navigation reflectors can be set arbitrarily in practical application, which can greatly reduce the complexity of setting the position of the reflector in the actual warehouse environment. The matching process of sequential comparison of distance and angle errors is cumbersome and inefficient, so the matching weight value w combining distance and angle error is proposed in this part.

In the localization process, if the distance range of the detected reflectors is too large, the distance error factor in the matching weight w will be dominant; if the range is too small, the angle error factor will play a leading role accordingly. Therefore, $\left(d_{near},d_{far}\right)$ is used to screen the appropriate reflector range to balance the error factor in the matching weight. Therefore, the navigation system screens out the n detected reflectors that are within the range of $\left(d_{near},d_{far}\right)$ from the robot based on the scanning results of the LiDAR to form a set of detected reflectors $ref_{n}$ and calculate the distance matrix $D_{n}$ and angle matrix $A_{n}$ between the robot and each detected reflector at this time.

In order to improve the calculation speed of the localization algorithm, it is also necessary to sort $D_{n}$ so that the navigation system can start to match the detected reflectors close to the robot. Since the motion compensation algorithm in the previous section can estimate the current position of the robot, the navigation system can filter out the m referential reflectors in the reflector map within the range of $\left(d_{near},d_{far}\right)$ from the estimated position to form the referential reflectors $ref_{m}$.The distance matrix $D_{m}$ and the angle matrix $A_{m}$ between the current position of the robot and each referential reflector are further calculated, and $D_{m}$ is sorted in the same way.

(22) $$D_{n}=\left[\begin{array}{c} d_{n1}\\ d_{n2}\\ d_{n3}\\ \vdots \\ d_{nn} \end{array}\right],A_{n}=\left[\begin{array}{c} \theta_{n1}\\ \theta_{n2}\\ \theta_{n3}\\ \vdots \\ \theta_{nn} \end{array}\right],D_{m}=\left[\begin{array}{c} d_{m1}\\ d_{m2}\\ d_{m3}\\ \vdots \\ d_{mm} \end{array}\right],A_{m}=\left[\begin{array}{c} \theta_{m1}\\ \theta_{m2}\\ \theta_{m3}\\ \vdots \\ \theta_{mm} \end{array}\right].$$

The matching weight value w of the navigation reflector is calculated and compared with the weight threshold $w_{\sigma }$ to filter out the matching reflector combination.

(23) $$w=\sigma_{d}ο\sigma_{a},$$

(24) $$\sigma_{d}=\left[\begin{array}{ccccc} d_{n1}-d_{m1} & d_{n2}-d_{m1} & d_{n3}-d_{m1} & \cdots & d_{nn}-d_{m1}\\ d_{n1}-d_{m2} & d_{n2}-d_{m2} & d_{n3}-d_{m2} & \cdots & d_{nn}-d_{m2}\\ d_{n1}-d_{m3} & d_{n2}-d_{m3} & d_{n3}-d_{m3} & \cdots & d_{nn}-d_{m3}\\ \vdots & \vdots & \vdots & \ddots & \vdots \\ d_{n1}-d_{mm} & d_{n2}-d_{mm} & d_{n3}-d_{mm} & \cdots & d_{nn}-d_{mm} \end{array}\right],$$

(25) $$\sigma_{a}=\left[\begin{array}{ccccc} \theta_{n1}-\theta_{m1} & \theta_{n2}-\theta_{m1} & \theta_{n3}-\theta_{m1} & \cdots & \theta_{nn}-\theta_{m1}\\ \theta_{n1}-\theta_{m2} & \theta_{n2}-\theta_{m2} & \theta_{n3}-\theta_{m2} & \cdots & \theta_{nn}-\theta_{m2}\\ \theta_{n1}-\theta_{m3} & \theta_{n2}-\theta_{m3} & \theta_{n3}-\theta_{m3} & \cdots & \theta_{nn}-\theta_{m3}\\ \vdots & \vdots & \vdots & \ddots & \vdots \\ \theta_{n1}-\theta_{mm} & \theta_{n2}-\theta_{mm} & \theta_{n3}-\theta_{mm} & \cdots & \theta_{nn}-\theta_{mm} \end{array}\right]$$

Taking the minimum value $w_{j,i}$ of the *i*-th column in w, if $w_{j,i}$ is within the matching weight threshold $w_{\sigma }$, it means that the *i*-th reflector in the detected reflector set $ref_{n}$ and the *j*-th reflector in the referential reflector set $ref_{m}$ are successfully matched.

### 2.5. The Calculation of Mobile Robot’s Position

To solve the localization issue with the artificial landmark approach, most algorithms use the trilateral localization method to calculate the position of the robot in the global coordinate system [30,31]. However, this approach has some drawbacks. When there is a measurement error present in reflector data, the three circles do not intersect at one point anymore, and the method will fail with a large error.

The particle filter is the technique to estimate the localization from a finite set of weighted random samples to approximate the posterior probability density of any particle state [32]. However, it heavily depends on the initial state, the number of particles used in calculation, and may have the convergence issue. For the navigation problem, except the position and angle, velocity, acceleration, and angular velocity could all be solved at the same time but require significant computing power. Quite different from particle filtering, the SVD algorithm only calculates the rotation vector and translation vector between the robot coordinate system and the global coordinate system with few computing resources. Therefore, SVD algorithm is used in this strategy to calculate the position of the robot in the global coordinate system.

During the matching process, the position matrix A of n detected reflectors and the position matrix B of n referential reflectors can be acquired; then, the rotation vector R and translation vector t of the position coordinate matrix can be calculated.

Where

B = RA + t.(26)

In order to calculate the rotation vector R and translation vector t, it is necessary to calculate the mean coordinates of two reflector position sets A and B; then, the mean coordinates of detected reflector position matrix A and referential reflector position matrix B are as follows:

(27) $$\left\{ \begin{array}{l} \mu_{A}=(x_{m_{A}},y_{m_{A}})=\frac{1}{n}\sum_{i=1}^{n}\left(x_{A_{i}},y_{A_{i}}\right)\\ \mu_{B}=(x_{m_{B}},y_{m_{B}})=\frac{1}{n}\sum_{i=1}^{n}\left(x_{B_{i}},y_{B_{i}}\right) \end{array}\right.,$$

$\mu_{A}$ and $\mu_{B}$ are equivalent to the central coordinates of the position matrix A and B. In order to calculate the rotation vector R, it is required to eliminate the influence of the translation vector t; therefore, the above reflector position matrix should be re-centered to generate the new reflector position matrix $A_{n}$ and $B_{n}$, and the covariance matrix H between the point sets should be calculated:

(28) $$\left\{ \begin{array}{l} A_{n_{i}}=[A_{i}-\mu_{A}]\\ B_{n_{i}}=[B_{i}-\mu_{B}]\\ H=\sum_{i=1}^{n}A_{n_{i}}B_{n_{i}}{}^{T}=\sum_{i=1}^{n}(A_{i}-\mu_{A}){\left(B_{i}-\mu_{B}\right)}^{T} \end{array}\right..$$

In the SVD algorithm, *U*, *S*, and *V* of matrix *H* can be obtained, and the optimized rotation vector *R* between *A* and *B* can be calculated:

(29) [U,S,V]=SVD(H),

(30) $$R=VU^{T}.$$

Ultimately, the translation vector t can be obtained by *R*:

(31) $$t=-R\mu_{A}+\mu_{B}.$$

**Figure 6:**
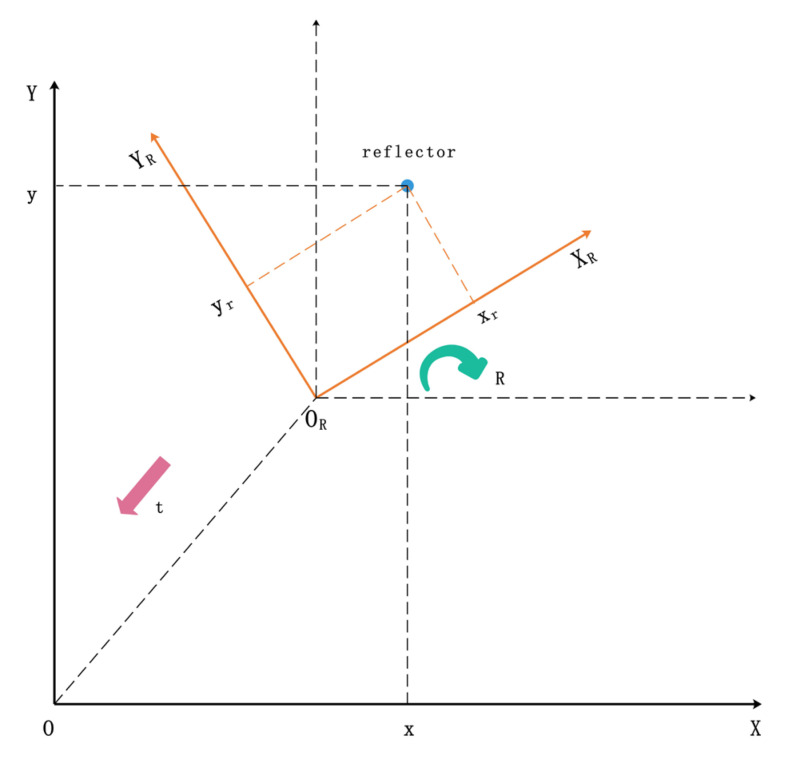
Rotation vector R and translation vector t.

The obtained rotation vector *R* and translation vector t are shown in Figure 6. The position coordinate of the robot in the robot coordinate system is $\left(x_{l},y_{l}\right)$, and the inverse operation is performed to obtain the position coordinate $\left(x_{g},y_{g}\right)$ in the corresponding global coordinate system:

(32) $$[x_{g},y_{g}]={\left(R^{-1}([x_{l},y_{l}]-t)\right)}^{T},$$

The orientation $\theta_{g}$ of the robot in the global coordinate system is as follows:

(33) $$\theta_{g}=-\arctan(\frac{R(2,1)}{R(1,1)})\cdot \frac{180^{\circ }}{\pi }.$$

## 3. Experimental Results

The scanning frequency of the Pepper-fuchs R2000 is set to 20 Hz. The R2000 has a field of view (FOV) of 360 degrees with the angular resolution of 0.25 degrees; detection range could reach 30 m with a nominal resolution of 1 mm and a maximum measured noise of 20 mm [33]. The SORLA navigation software runs on the embedded computer with i5-6200u CPU (2.3 GHz) and 4 GB mem. During the runtime, the average CPU usage is about 12%, and the average physical mem usage is 25%.

To fully test the robustness of reflector matching, failure test cases are designed and the errors such as reflector displacement are created. The algorithm is also examined for some extreme cases in which some reflectors are blocked. It can be verified by the following experiments that the localization accuracy of the navigation system in this work reaches a high level for the practical industrial applications, and the system still performs well when the robot is moving at high speed or the reflectors have small displacement.

### 3.1. Localization Accuracy in Initialization Mode

**Figure 7:**
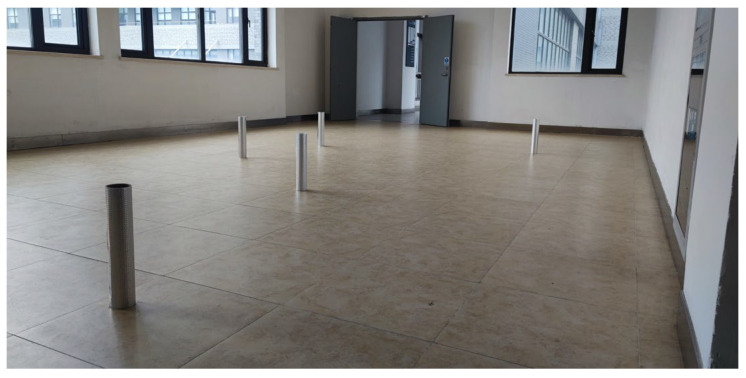
The experimental layout environment of 5x initialization reflectors.

In a practical warehouse, a forklift or AGV always starts the navigation from the initial zone. The initialization mode is designed to calibrate the initial position shifts caused by misalignment from warehouse operation and make the system more robust to operate. In this experiment, three cases with different numbers of initialization reflectors were set to verify the effect of the number of initialization reflectors on localization accuracy in the initialization mode and localization accuracy in each case; the number is set to 3, 4, and 5 respectively. The global coordinates of reflectors are (−1744, −652), (1854, −1838), (784, 676), (−636, 757), and (−3850, −1335), with the unit of mm. The experimental environment is shown in Figure 7.

**Figure 8:**
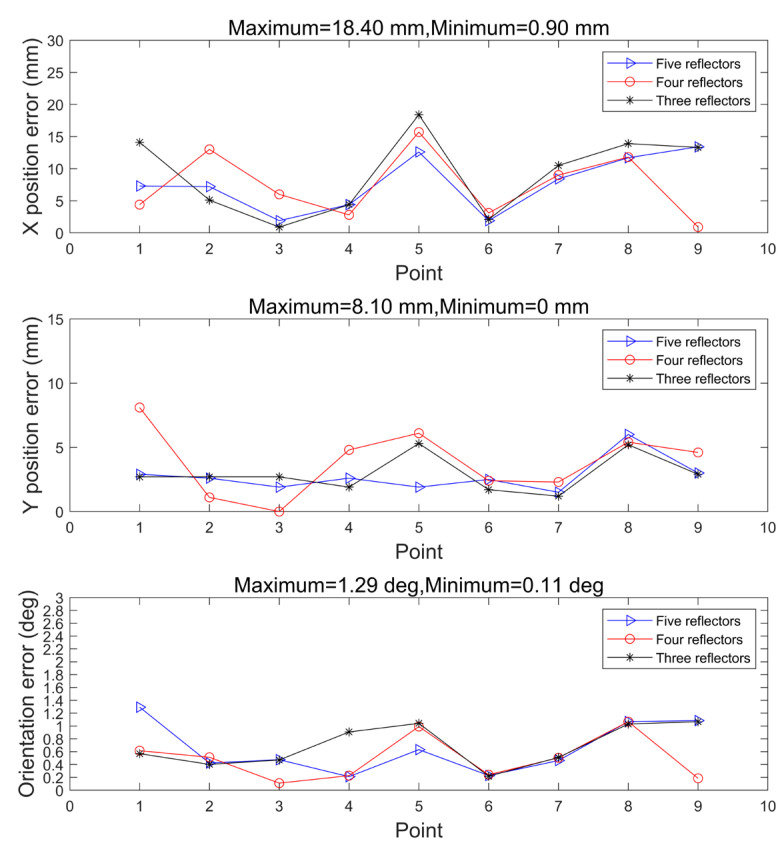
Initial point localization error in initialization mode.

Localization data are obtained at nine different locations. The position error and orientation angle error obtained in the three cases are shown in Figure 8. It can be seen that the localization accuracy of the initial point is not necessarily related to the number of initialization reflectors, and it is not true that the more the number of reflectors, the higher the localization accuracy of the initial point. The experimental results show that the maximum X-direction localization error of the initial position is 18.4 mm, the maximum Y-direction localization error is 8.1 mm, and the maximum angle error is 1.29 deg, which meets the localization accuracy requirements of industrial application environment; the localization error statistics in X- and Y- directions are shown in Table 1 and Table 2, and the angle error statistics are shown in Table 3.

The optimal triangular positioning method based on angle measurement also uses five reflectors to perform static positioning at nine different positions [16]. Table 4, Table 5 and Table 6 are the comparisons of the positioning method in initialization mode and the optimal triangular positioning method in the X-direction positioning error, the Y-direction positioning error, and the orientation error, respectively. The localization method in this study shows more accurate results of the X -direction and angle. It is worth mentioning that even with such high resolution, the maximum noise of 20 mm will be converted to the same amount of variation to LiDAR data and contaminate the localization precision. By applying the proposed strategy, the localization precision can be improved to 6 mm very stably, which is one of the best localization results of AGV both for academy and industry.

### 3.2. The Influence of Motion Speed on Navigation Accuracy

**Figure 9:**
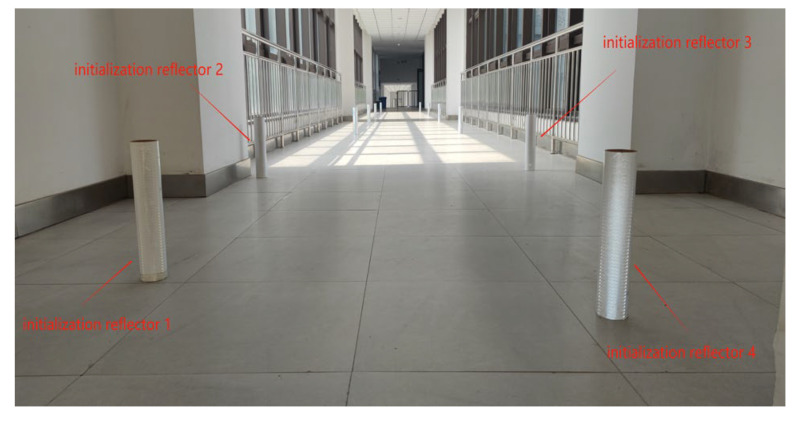
The actual layout environment of reflectors.

**Figure 10:**
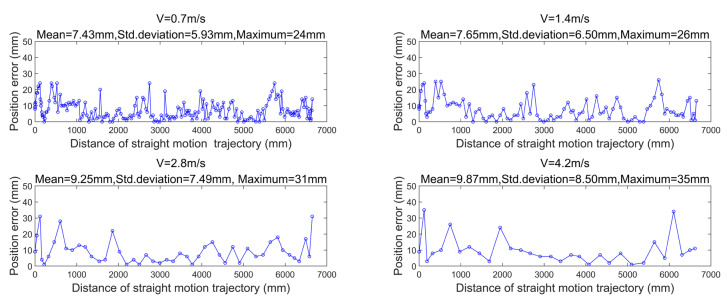
Localization accuracy at different speeds.

In this part, we demonstrate the performance of the algorithm proposed in Section 2.4.1. This experiment is designed to verify the localization accuracy of the system in high-speed movement. First, four initialization reflectors and eight navigation reflectors are set up along a corridor, as shown in Figure 9. The localization error results are shown in Figure 10; the robot moves along the straight line at the speed of 0.7, 1.4, 2.8, and 4.2 m/s, respectively. The location estimation error and the reflector matching error become large in the case of high-speed movement, so results indicate that the average error and maximum error of localization increase with the movement speed. The location error caused by faster speed is well compensated with the help of motion compensation. Therefore, the navigation system is quite durable for the robot under high-speed movement, and the localization accuracy remains at a high level.

### 3.3. The Influence of Motion Compensation Algorithm on High-Speed Turning

**Figure 11:**
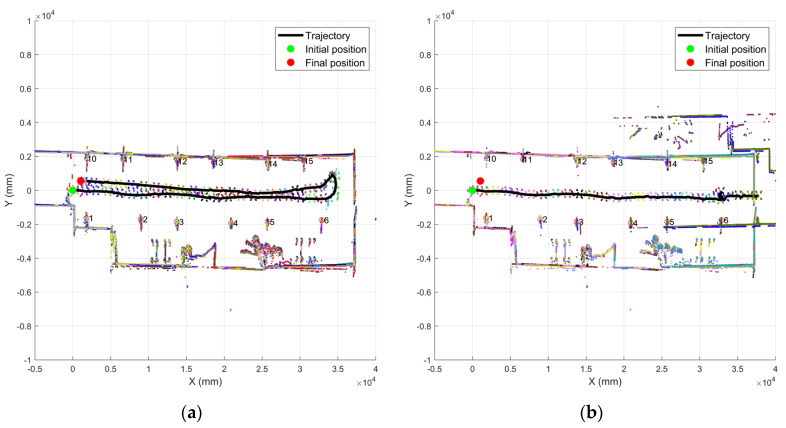
Robot motion trajectory and navigation map: (**a**) motion compensation mode turned on; (**b**) motion compensation mode turned off.

Since the navigation system has a motion compensation algorithm to accurately estimate the position of the robot at the next moment, it can achieve the accurate location under high-speed movement and also supports accurate localization for the case of fast turning. Figure 11 shows the accumulated robot’s path and the converted navigation map for each movement. The locations of reflectors are all marked with the identification numbers. When the motion compensation mode is enabled, the trajectory of the robot movement is shown in Figure 11a with a speed of 2.6 m/s, and the robot makes a sharp turn with the angular velocity of 1.4 rad/s. There is no navigation failure happened caused by angular mismatch during the navigation. When the motion compensation mode is disabled, the robot moves along the path with the same speed, and the navigation system has a large localization error around the corner, which results in the navigation failure, as shown in Figure 11b. Therefore, the experimental result has verified that the motion compensation algorithm can handle the large angle velocity properly, which is critical for forklift operation in the warehouse.

### 3.4. The Influence of Relative Displacement of the Reflector on Navigation Accuracy

**Figure 12:**
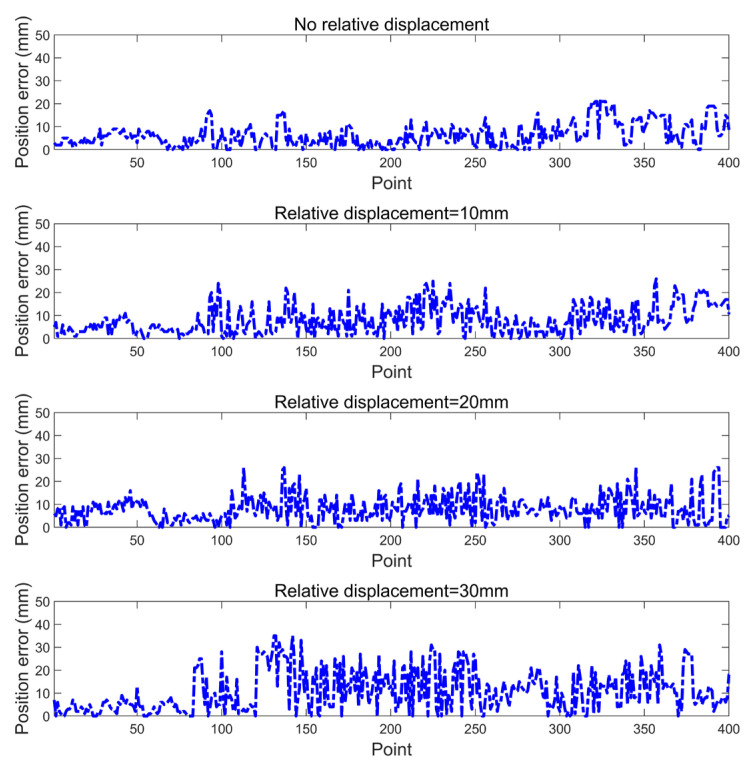
Localization error of the navigation reflectors with relative displacement.

Due to human disoperation and some random errors in the warehouse environment, the reflector installation location may vary from the original locations in the map. The navigation system in this algorithm is designed to complete accurate localization when the displacement is presented in the reflector layout. This experiment is designed to investigate the algorithm’s capability to handle the reflector displacement. The systematic displacement is created for the navigation reflectors in the navigation map from the experimental settings; to observe the influence of the relative displacement of the navigation reflectors on the localization accuracy, the relative displacement of 0, 10, 20, and 30 mm of each navigation reflector is set, respectively, to allow the robot to move along the same straight line at a speed of 0.4 m/s. The layout of reflectors is shown in Figure 9. The localization error results are shown in Figure 12 and Table 7; it can be seen from the results that with the increase of the relative displacement between the ideal position and the actual position of the navigation reflectors, the navigation localization accuracy will decrease slightly. This shows that the navigation system has a strong resistance to the error of reflector location.

### 3.5. Validation of Navigation Recover Mechanism

In the actual navigation process of mobile robots, they often encounter situations in which the navigation fails because of the obstruction of the reflectors. When the navigation failure happens, the system will switch to the initialization mode and recalculate the current position of the robot; if the calculation is successful, the system will recover and resume the navigation mode from a new location.

**Figure 13:**
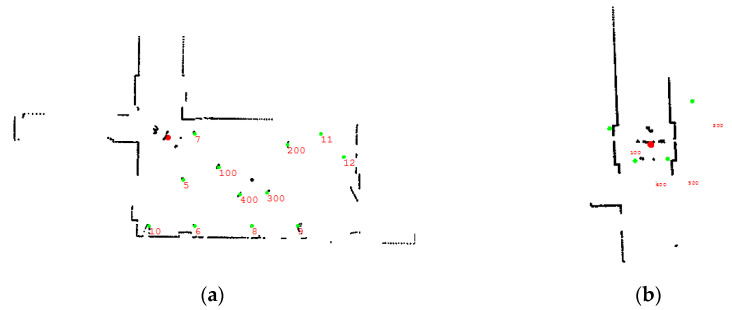
System real-time navigation map: (**a**) normal navigation mode; (**b**) navigation mode failure in the corridor.

**Figure 14:**
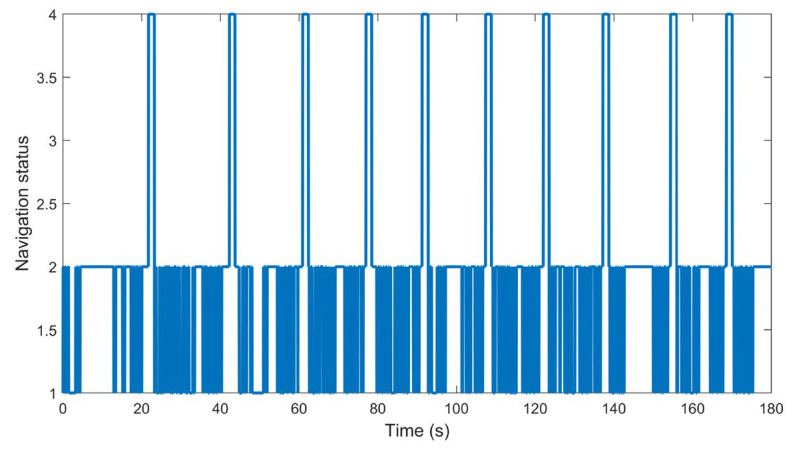
Navigation status change trend.

The navigation system determines the navigation status and outputs the navigation status value through the CAN Bus Analyzer. According to the root mean square (RMS) error of reflector matching results, the navigation system decides the navigation status in four levels: status 1, 2, 3, and 4. Status 1 refers to the RMS error between the ideal location and the actual location of the reflector being between 1 and 10, status 2 refers to the RMS being between 10 and 100, status 3 refers to the RMS being between 100 and 500, and navigation status 4 refers to the navigation failure and the RMS being above 1000. During the experiment, there is a corridor next to the experimental site where the reflector is installed. The robot moves back and forth between the corridor and the experimental site 10 times. When the mobile robot enters the corridor, the laser scanner cannot detect the reflectors, because the reflectors are blocked by the wall, which eventually causes the navigation failure. If the robot returns to the experimental site, the system will restart the navigation mode again. The actual scene of the system scanning is shown in Figure 13, and the actual size of the rectangular experimental area in Figure 13 is 11.4 × 6 m. The navigation status data collected by the system are shown in Figure 14. It can be seen that the robot can successfully restart the navigation mode after each navigation failure.

### 3.6. System Stability Test

**Figure 15:**
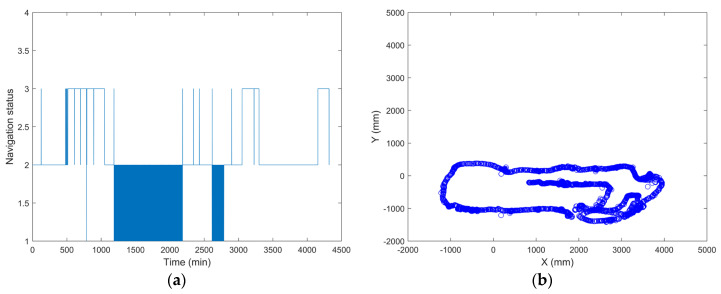
System stability experiment for long-term operation: (**a**) navigation status; (**b**) navigation trajectory curve with time elapse.

In addition, the navigation system can work effectively and robustly for a long time. The CAN Bus Analyzer outputs the navigation status data, and the system can run continuously for 72 h. During the experiment, the mobile robot randomly moves to a new location every 2 h. The experimental results show that the navigation status is basically maintained at 1 and 2, sometimes it jumps to 3, and there no navigation failure happened. This indicates that the navigation and the memory usage of the system are able to run for a long time in a relatively stable situation. The navigation status and trajectory curves during the experiment are shown in Figure 15.

## 4. Conclusions

In this work, the localization strategy with cylindrical reflectors and laser-data only has been introduced in great detail. The SORLA algorithm has shown high precision, strong robustness, low requirements on the computing hardware, and “real-time” localization with high-speed moving and fast-turning capability. SORLA is designed to operate in two successive operational modes. (1) In initialization mode, the reflector layout are defined by the distinctive geometrical relationship. This mode is required to initialize the robot location before the navigation task or loses track on the location and needs to relocate. (2) In navigation mode, the reflector layout could be deployed in the large area without the limitation of the geometric relationship. This is feasible due to the fact that the probability of the LiDAR locations of multiple “features” could be solved by the SVD algorithm. By introducing the reflector extraction, motion compensation, and reflector matching mechanism, the algorithm only needs LiDAR data to complete the high-precision localization without the help of any other sensors. Since there may be errors in the installation of the navigation reflector, we design the positive and negative experiments, and the results show the algorithm can handle the reflector displacement or the reflector occlusion relatively well.

According to the experimental results, the localization error of the navigation system is about 6.45 mm for the speed of 0.4 m/s and 9.87 mm when the speed reaches 4.2 m/s, and still works well with the angular velocity of 1.4 rad/s at turn, which is suitable for real-time localization requirements at high speed or fast turning. Although our strategy is durable for laser navigation with a lightweight computing workload, it is possible that the “feature extraction” part could be replaced by other techniques, such as the laser contour profile or visual segmentation solution, and this makes the strategy workflow a more promising alternative to Laser SLAM or Visual SLAM.

## Figures and Tables

**Table 1:** X position error.

The Number of Reflector	Mean (mm)	Maximum (mm)	Std. Deviation (mm)
3	9.05	18.4	5.67
4	7.64	15.7	4.69
5	7.89	13.4	3.96

**Table 2:** Y position error.

The Number of Reflector	Mean (mm)	Maximum (mm)	Std. Deviation (mm)
3	3.52	5.3	2.21
4	4.12	8.1	2.44
5	3.36	6.0	2.13

**Table 3:** Orientation error.

The Number of Reflector	Mean (deg)	Maximum (deg)	Std. Deviation (deg)
3	0.64	1.07	0.32
4	0.48	1.07	0.32
5	0.62	1.29	0.37

**Table 4:** The comparison in X position error.

Method	Mean (mm)	Maximum (mm)
Optimal triangulation positioning algorithm based on angle measurement	13.34	17.58
Positioning algorithm in initialization mode	7.89	13.4

**Table 5:** The comparison in Y position error.

Method	Mean (mm)	Maximum (mm)
Optimal triangulation positioning algorithm based on angle measurement	2.2	6.0
Positioning algorithm in initialization mode	3.36	6.0

**Table 6:** The comparison in orientation error.

Method	Mean (deg)	Maximum (deg)
Optimal triangulation positioning algorithm based on angle measurement	0.89	1.81
Positioning algorithm in initialization mode	0.62	1.29

**Table 7:** Localization error of the navigation reflectors with relative displacement.

Relative Displacement (mm)	Mean (mm)	Maximum (mm)	Std. Deviation (mm)
0	6.45	22	5.08
10	8.82	26	6.37
20	8.84	26	6.27
30	12.17	35	9.11

## Data Availability

The MATLAB code of the SORLA presented in this study is available in [https://github.com/unlogical0327/RLA_V15_Implementation] (accessed on 21 January 2019). Video clips captured for an unmanned forklift and SORLA software screen record could be found at [https://github.com/unlogical0327/SORLA] (accessed on 24 May 2021).
